# Capacity and limitations of microfluidic flow to increase solute transport in three-dimensional cell cultures

**DOI:** 10.1098/rsif.2024.0463

**Published:** 2025-01-29

**Authors:** Willy V. Bonneuil, Neeraj Katiyar, Maria Tenje, Shervin Bagheri

**Affiliations:** ^1^ Department of Engineering Mechanics, KTH Royal Institute of Technology, Stockholm, Sweden; ^2^ Department of Materials Science and Engineering, Uppsala University, Uppsala, Sweden; ^3^ Science for Life Laboratory, Uppsala University, Uppsala, Sweden

**Keywords:** organ-on-chip, three-dimensional cell culture, solute transport

## Abstract

Culturing living cells in three-dimensional environments increases the biological relevance of laboratory experiments, but requires solutes to overcome a diffusion barrier to reach the centre of cellular constructs. We present a theoretical and numerical investigation that brings a mechanistic understanding of how microfluidic culture conditions, including chamber size, inlet fluid velocity and spatial confinement, affect solute distribution within three-dimensional cellular constructs. Contact with the chamber substrate reduces the maximally achievable construct radius by 15%. In practice, finite diffusion and convection kinetics in the microfluidic chamber further lower that limit. The benefits of external convection are greater if transport rates across diffusion-dominated areas are high. Those are omnipresent and include the diffusive boundary layer growing from the fluid–construct interface and regions near corners where fluid is recirculating. Such regions multiply the required convection to achieve a given solute penetration by up to 100, so chip designs ought to minimize them. Our results define conditions where complete solute transport into an avascular three-dimensional cell construct is achievable and applies to real chambers without needing to simulate their exact geometries.

## 1. Introduction

The morphology and behaviour of living cells fundamentally change with the dimensionality of the environment they grow in. Three-dimensional cell cultures more accurately replicate the conditions that cancerous cells, migrating cells (e.g. immune cells) [1,2] or stem cells [3] experience *in vivo* [4]. The understanding of organ development and tumour growth has been furthered by cell-culture techniques that promote the self-assembly of cells into spheroids or organoids (stem-cell-derived three-dimensional tissue constructs) and where biological processes can be modulated by controlled physicochemical stimuli [5–7]. These techniques also show promise to accelerate drug testing [8]. Here, we define three-dimensional tissue constructs grown in controlled environments as multi-cellular engineered living systems (M-CELS). M-CELS acquire a broader physiological relevance as they grow larger because that lets them mimic more advanced development stages. Most M-CELS are engineered without a perfusable vasculature, which induces diffusion-limited growth. The incorporation of perfusable vasculatures into M-CELS is actively researched, but is complex and lacks a generally applicable strategy [9–11]. Nutrient transport and waste removal in avascular M-CELS rely on diffusion from their surface. Cells beyond a certain depth hence do not receive enough nutrients and become necrotic, i.e. they prematurely die. Necrosis affects the phenotype of the remaining live cells, e.g. by releasing inflammatory or chemo-attractant markers into the intercellular space [12] and altering the mechanical properties of the live rim [13]. The balance between proliferating cells on the surface of M-CELS and dying cells at their centre confers M-CELS a diffusion-limited maximum diameter [14]. Both phenotype alteration and size limitation restrict the stage of tumour growth or organ development up to which avascular M-CELS are a relevant biological model.

Microfluidic technology has enabled the culture of avascular M-CELS of larger diameters and greater compartmentalization, i.e. of more advanced development [15–18]. The fluid velocities over M-CELS vary widely, as have the M-CELS-confining structures which are necessary for their retention. The magnitudes of the improvements brought about by microfluidic culture have been mixed. In one brain-organoid study, organoid diameters increased by a fifth under a steady fluid flow of 0.1 mm s^−1^ (our calculation, from parameters listed in the paper), without necrotic-core prevention [15]. In another brain-organoid study, diameters increased by a third under the oscillatory fluid flow of 1 mm s^−1^ (our calculation), with necrotic-core prevention [18]. Fluid flow has been treated as an ‘on–off’ parameter in many studies comparing static with microfluidic culture. Its varying effects encourage the mechanistic elucidation of its influence on solute transport in M-CELS.

Previous modelling efforts on nutrient transport into M-CELS have yielded analytical expressions for nutrient concentration in systems with spherical [19,20] or ellipsoidal [21] symmetry, isolated from their culture environment. Numerical work has been conducted on nutrient supply to spheroids in specific culture set-ups, including culture traps [22] or confinement pillars [23]. The dimensions of defined chamber geometries have been optimized to increase nutrient supply to M-CELS of a given cell type [24,25]. These numerical studies have not established general principles of chamber design and operation by which to harness the benefits of microfluidic flow and which can be quantitatively applied at the experiment-design stage. Here, we achieve this by combining theoretical and numerical analyses of nutrient supply to M-CELS. This study accounts for the distance between M-CELS and a nutrient source and M-CELS structural confinement. It makes the influence of chamber geometry and flow rate on solute transport and necrosis occurrence more predictable. Although nutrient supply is a primary interest, this investigation applies to any solutes, including drugs, that diffuse into an M-CELS and whose freely diffusing form has a sink term: consumption or binding to an agonist receptor. We estimate not only the conditions for the prevention of necrosis, but more generally those for an increase in penetration distance. Our results highlight the penalizing role of large diffusion-dominated areas such as corners or hydrogel capsules and of fast diffusion within M-CELS, which both delay the solute-transport enhancement brought by convection.

## 2. Methods

### 2.1. Computational domains

Let a computational domain Ω comprise an M-CELS as an inclusion $\Omega_{2}$ in a straight fluid channel $\Omega_{1}$, such that $\Omega_{1}\cup \Omega_{2}=\Omega$. The interface between the domains is noted ${\bar{\Omega }}_{1}\cap {\bar{\Omega }}_{2}=\Gamma$. The fluid channel has an inlet I and an outlet O. The channel walls were noted $W_{0}$ at y=0 and $W_{h}$ at y=2h and their union W. $\Omega_{2}$ was assumed porous and rigid with homogeneous properties (table 1).

**Table 1.** Symbol table.

symbol	meaning
$\Omega_{1}$	microfluidic channel
$\Omega_{2}$	M-CELS
${\hat{\Omega }}_{i}$	domain i in the radial-linear analytical geometry
Γ	fluid–M-CELS interface
I	channel inlet
O	channel outlet
W	wall boundary
S	symmetry boundary
v	fluid velocity
p	fluid pressure
$v_{0}$	peak inlet fluid velocity
a	M-CELS radius
L	distance between M-CELS and channel inlet
k	Darcy permeability (homogeneous to a surface area)
ρ	fluid density
μ	dynamic viscosity
c	solute concentration
$c_{0}$	inlet solute concentration
$D_{i}$	solute diffusivity in domain $\Omega_{i}$
$R_{\max}$	maximum solute consumption rate
$\Phi_{L}$	fraction of cells receiving enough solute in $\Omega_{2}$
subscript 0	relative to the no-fluid-flow asymptote
subscript ∞	relative to the maximal-supply asymptote

**Figure 1.**
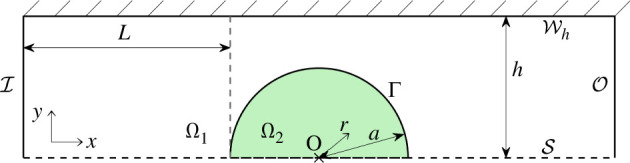
Computational domain (unconfined). M-CELS as a disc $\Omega_{2}$ of radius a in a rectangular channel $\Omega_{1}$. Ω bisected by a symmetry line S, parallel to the direction of flow. Coordinate origin at the centre of the M-CELS.

**Figure 2.**
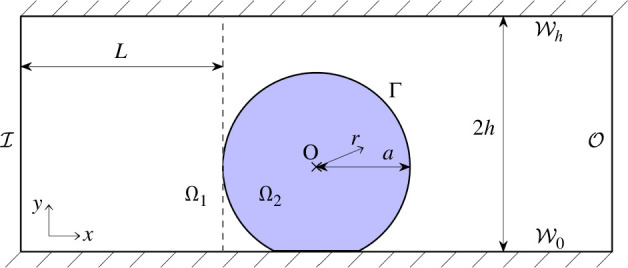
Computational domain (confined). M-CELS as a partial disc $\Omega_{2}$ cut such that its height above the bottom wall of the fluid channel is 90% of its diameter. Coordinate origin at the centre of the smallest circle containing $\Omega_{2}$.

To isolate the effects of M-CELS confinement, we simulated two geometries. First, an ‘unconfined’ geometry where $\Omega_{2}$ is a disc surrounded by fluid (figure 1) and where Ω is bisected by a symmetry line S. The flow and the M-CELS are confined by the channel walls. The term ‘unconfined’ refers to the M-CELS having no contact with any solid. Second, a confined geometry where $\Omega_{2}$ is a disc cut such that its height from the bottom of the channel is 90% of its diameter (figure 2). $\Omega_{2}$ is in contact with $W_{0}$ through its linear boundary. This confinement is ‘geometrically minimal’ in that it includes fluid-recirculation areas upstream and downstream of obstacles (in $\Omega_{1}$ close to the contact points $\Omega_{1}\cap \Omega_{2}\cap W$) and an area of contact between the M-CELS and a solid wall, but does not include wall-normal obstacles. The surface area of contact $\Omega_{2}\cap W_{0}$ corresponds to the one observed in an experimental human lung-fibroblast spheroid (PB-CH-450-0811, PELOBiotech, Germany) cultured between confinement pillars in a microfluidic channel (electronic supplementary material, figure S1). In both geometries, Ω is described by a Cartesian coordinate system (x,y) centred on the centre O of the smallest circle containing $\Omega_{2}$. A radial coordinate system (r,θ) is also defined from O.

### 2.2. Quasi-one-dimensional analytical domain

**Figure 3.**
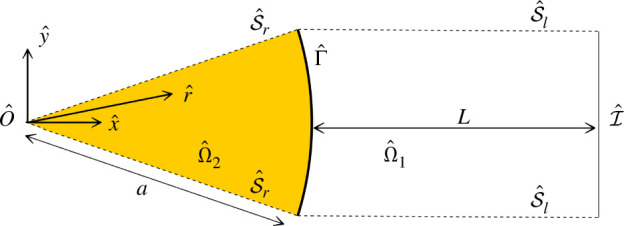
Quasi-one-dimensional ‘radial-linear’ reduction of Ω into $\hat{\Omega }$. The angular section ${\hat{\Omega }}_{2}$ has an angle dθ≪1.


To acquire an analytical understanding of the problem, we define a quasi-one-dimensional ‘radial-linear’ domain $\hat{\Omega }$ as the union of a radial section of a disc of radius a and angle dθ, named ${\hat{\Omega }}_{2}$ and centred on $\hat{O}$, and the constant-width extension of that section, named ${\hat{\Omega }}_{1}$ (figure 3). $\hat{\Gamma }$ and $\hat{I}$ and the coordinate systems $\left(O,\hat{x},\hat{y}\right)$ and $\left(O,\hat{r},\hat{\theta }\right)$ are defined in analogous ways to their two-dimensional counterparts. The planes ${\hat{S}}_{r}=\left\{\hat{\theta }=\pm \;d\theta /2\right\}$ are symmetry planes for ${\hat{\Omega }}_{2}$ and the planes ${\hat{S}}_{l}$ of normal $\hat{y}$ that bound ${\hat{\Omega }}_{1}$ are symmetry planes for ${\hat{\Omega }}_{1}$. If dθ≪1, we assume that, for a scalar ξ

(2.1) $${\frac{d\xi }{d\hat{r}}\cdot \hat{r}|}_{\hat{\Gamma }}={\frac{d\xi }{d\hat{x}}\cdot \hat{x}|}_{\hat{\Gamma }}.$$

The assumptions of symmetry and narrowness of ${\hat{\Omega }}_{2}$ allow equations in $\hat{\Omega }$ to be reduced to a quasi-one-dimensional ‘radial-linear’ form where variables depend only on $\hat{x}$ in ${\hat{\Omega }}_{1}$ and only on $\hat{r}$ in ${\hat{\Omega }}_{2}$.

### 2.3. Equations for fluid flow

Fluid flow in $\Omega_{1}$ is modelled by the steady Navier–Stokes equations with a parabolic inlet velocity and normal outlet flow (electronic supplementary material, Section M1). The normalized momentum conservation equation is


(2.2) $$Re(v_{1}^{*}\cdot \nabla^{*})v_{1}^{*}=-\nabla^{*}p^{*}+\nabla^{*}\cdot (\nabla^{*}v_{1}^{*}+\nabla v_{1}^{*T}),$$

where the Reynolds number has been defined as $Re=\rho v_{0}a/\mu$, local acceleration has been neglected, and non-dimensional variables are noted with asterisks. Microfluidic conditions involve peak inlet velocities of the order of 1 mm s^−1^, M-CELS radii are at most of the order of 1 mm, and cell culture medium has similar density and viscosity to those of water [26], so Re≲1. We therefore did not neglect convective acceleration.

Fluid flow in $\Omega_{2}$ is modelled by the Darcy equations,


(2.3a) $$\begin{array}{r} 0=\frac{k}{\mu }\nabla p_{2}+v_{2} \end{array},$$

(2.3b) $$\begin{array}{rl} \nabla \cdot v_{2} & =0 \end{array}.$$

We assumed that the M-CELS had homogeneous properties, which is a valid assumption at least at the early stages of M-CELS growth when little cellular differentiation has occurred. Continuity of pressure and velocity are prescribed at the fluid–porous interface,


(2.4a) $$\begin{array}{rl} p_{1} & ={p_{2}|}_{\Gamma }, \end{array}$$

(2.4b) $$\begin{array}{rl} v_{1} & ={v_{2}|}_{\Gamma }. \end{array}$$

Pressure continuity may be applied at a fluid–porous interface when there is a separation of scales in the porous domain (here, the inter-cell distance of approximately 10 nm is much smaller than the M-CELS radius of at least 100 µm [27]). The continuity of velocity between its Navier–Stokes and Darcy solutions results from mass conservation and the assumption that the slip length in $\Omega_{2}$ is zero. The latter scales with $\sqrt{k}$ so is at most 100 nm and therefore, much smaller than a cell (M-CELS are expected to be less permeable than the most permeable tissues, where $k∼10^{-14}\;m^{2}$ [28]).

### 2.4. Equations for nutrient transport

Solute transport is simulated by steady advection–diffusion–reaction equations. For i∈{1,2},


(2.5) $$0=\nabla_{i}\cdot (D_{i}\nabla c_{i})-\nabla_{i}\cdot (v_{i}c_{i})-R(c_{i}).$$

The velocities $v_{i}$ are obtained from the fluid-flow equations. Diffusivities are uniform in each domain, at $D_{i}$. Solute consumption is 0 in $\Omega_{1}$ and follows Michaelis–Menten kinetics in $\Omega_{2}$, as in models of tumour spheroids [23,29],


(2.6) $$R(c_{2})=\frac{R_{max}\;c_{2}}{c_{2}+K_{1/2}}.$$

The consumption rate increases continuously from 0 in the absence of solute to $R_{\max}$ at high solute concentration ($c_{2}\gg K_{1/2}$). The half-rate constant $K_{1/2}$ verifies $R(K_{1/2})=R_{\max}/2$. The consumption parameters are uniform in the M-CELS, which is reasonable at the early stages of development. An unlimited solute supply, $c=c_{0}$, was assumed at I and O. This represents the common placement of a culture-medium reservoir on either side of a microfluidic chamber.

Let non-dimensionalized variables, noted with asterisks, be defined as $x_{1}=x_{1}^{*}L$, $x_{2}=x_{2}^{*}a$, $v_{1}=v_{1}^{*}v_{0}$ and $c_{i}=c_{i}^{*}c_{0}$ for i∈{1,2}. To non-dimensionalize $v_{2}$, we combine the pressure drop across a sphere in Stokes’ flow ($\Delta p_{S}∼\mu v_{0}/a$) and Darcy’s law ($v_{2}∼(k/\mu )(\Delta p_{S}/a)$) to define $v_{2}=v_{2}^{*}kv_{0}/a^{2}$. We also define $r=r^{*}a$ and $K_{1/2}=K_{1/2}^{*}c_{0}$. In $\Omega_{1}$, the non-dimensionalized transport equation is


(2.7) $$0=\nabla_{1}^{*2}c_{1}^{*}-{Pe}_{1}\nabla^{*}\cdot (v_{1}^{*}c_{1}^{*}),$$

where the Péclet number is ${Pe}_{1}=v_{0}L/D_{1}$. In $\Omega_{2}$, the non-dimensionalized transport equation is


(2.8) $$0=\nabla_{2}^{*2}c_{2}^{*}-{Pe}_{2}\nabla \cdot (v_{2}^{*}c_{2}^{*})-Da\frac{c_{2}^{*}}{c_{2}^{*}+K_{1/2}^{*}},$$

where the Péclet number is ${Pe}_{2}=kv_{0}/(aD_{2})$ and the Damköhler number is $Da=R_{\max}a^{2}/(c_{0}D_{2})$.

The most favourable situation for convection in $\Omega_{2}$ shows the latter is negligible. Microfluidic flows rarely have a peak velocity higher than 1 mm s^−1^, the permeability of M-CELS is assumed under 10^−14^ m^2^ (that of the most permeable tissues [28]), and the question of solute transport sufficiency in M-CELS usually arises when those are larger than 100 μm. The Stokes–Einstein diffusivity in water of solutes of interest in M-CELS development is above $10^{-11}\;m^{2}\;s^{-1}$ (which is the value for proteins of 100 kDa). We assume that $D_{2}/D_{1}>0.01$ for non-lipophilic solutes (those that do not cross cellular membranes), so $D_{2}>10^{-13}\;m^{2}\;s^{-1}$. The value of 0.01 is the order of the lowest effective glucose diffusivity over a range of tumour spheroids [30]. This yields ${Pe}_{2}<0.1$, so solute transport in $\Omega_{2}$ may be described by a diffusion–reaction equation,


(2.9) $$0=\nabla_{2}^{*2}c_{2}^{*}-Da\frac{c_{2}^{*}}{c_{2}^{*}+K_{1/2}^{*}}.$$

A no-flux condition is prescribed on the symmetry plane and walls,


(2.10) $${\left(\nabla c_{i}\cdot n\right)|}_{W\cup S}=0,\;i\in \{1,2\}.$$

At the interface Γ, conservation of nutrient quantity implies continuity of concentration. Let the interface concentration be noted γ,


(2.11) $${c_{1}|}_{\Gamma_{1}}={c_{2}|}_{\Gamma_{2}}=\gamma .$$

The evaluation of a quantity on $\Gamma_{i}$ is understood as the limit of that quantity when approaching Γ from $\Omega_{i}$. Conservation of nutrient quantity also implies continuity of normal diffusive fluxes,


(2.12) $$D_{1}{\frac{\partial c_{1}}{\partial n}\cdot n|}_{\Gamma_{1}}=D_{2}{\frac{\partial c_{2}}{\partial n}\cdot n|}_{\Gamma_{2}},$$

with n the normal to Γ (of arbitrary orientation).

### 2.5. Nutrient balance at the fluid–multi-cellular engineered living systems interface

Three additional non-dimensional quantities are defined to describe physical balances of importance on Γ.

The ratio of diffusive solute fluxes along the normal n (oriented into $\Omega_{2}$) is


(2.13) $$\begin{array}{rl} \frac{J_{1}}{J_{2}} & =\frac{-D_{1}{\frac{\partial c_{1}}{\partial n}\cdot n|}_{\Gamma_{1}}}{-D_{2}{\frac{\partial c_{2}}{\partial n}\cdot n|}_{\Gamma_{2}}}\\ & =\frac{a\;D_{1}}{L\;D_{2}}\frac{\frac{\partial c_{1}^{*}}{\partial n^{*}}\cdot n^{*}}{\frac{\partial c_{2}^{*}}{\partial n^{*}}\cdot n^{*}}. \end{array}$$

This ratio is equal to 1, so the ratio of interface concentration gradients is characterized by


(2.14) $$\frac{\frac{\partial c_{2}^{*}}{\partial n^{*}}\cdot n^{*}}{\frac{\partial c_{1}^{*}}{\partial n^{*}}\cdot n^{*}}=\frac{aD_{1}}{LD_{2}}.$$

Let us define


(2.15) $$R_{d}=\frac{aD_{1}}{LD_{2}},$$

which characterizes whether diffusion into $\Omega_{2}$ is supply-limited (low $R_{d}$) or rate-limited (high $R_{d}$).

Let us approximate γ as uniform to consider the solute quantity balance in a radial section of $\Omega_{2}$ of angle dθ centred on O. The corresponding section of Γ receives


(2.16) $$n_{d}=-D_{1}ad\theta \frac{dc_{1}}{dn}\cdot n$$

by diffusion and


(2.17) $$n_{c}=ad\theta \gamma v_{1}\cdot n$$

by convection. Let $r_{n}$ be the radius where $c_{2}$ falls to zero. In a radially symmetric geometry, $\Gamma_{n}$ is the circle $r=r_{n}$. We allow $r_{n}=0$ if $c_{2}$ is positive everywhere. The radial section loses the following solute quantity due to consumption:


(2.18) $$\begin{array}{rl} n_{R} & =R_{max}\int_{r_{n}}^{a}rd\theta dr\\ & =\frac{R_{max}}{2}(a^{2}-r_{n}^{2})d\theta . \end{array}$$

Let us now normalize the solute quantities and consider the ratio between diffusive supply to Γ and consumption in $\Omega_{2}$:


(2.19) $$\frac{n_{d}}{n_{R}}=\frac{2D_{1}c_{0}}{R_{max}aL}\frac{1-r_{n}^{*2}}{\frac{dc_{1}^{*}}{dn}\cdot n},$$

from which we define the diffusive supply number as


(2.20) $$S_{d}=\frac{2c_{0}D_{1}}{R_{max}aL}.$$

$S_{d}$ describes how the diffusive supply of solutes to Γ affects the quantity of solutes consumed in $\Omega_{2}$. If $S_{d}<1$, solute consumption is supply-limited, i.e. the quantity consumed by the M-CELS is limited by the quantity that it receives from its environment. If $S_{d}>1$, solute consumption is rate-limited, i.e. the M-CELS consumes as much as its biology demands. $S_{d}$ may be expressed as


(2.21) $$S_{d}=\frac{2R_{d}}{Da}.$$

Let us consider the ratio between convective solute supply to Γ and consumption in $\Omega_{2}$,


(2.21) $$\frac{n_{c}}{n_{R}}=\frac{2c_{0}v_{0}}{R_{max}a}\frac{1-r_{n}^{*2}}{\gamma^{*}v_{1}^{*}\cdot n},$$

from which we define the convective supply number as


(2.23) $$S_{c}=\frac{2c_{0}v_{0}}{R_{max}a}.$$

$S_{c}$ may be described in an analogous way to $S_{d}$ and expressed as


(2.24) $$S_{c}=\frac{2R_{d}{Pe}_{1}}{Da}.$$

The ratios $R_{d}$, $S_{d}$ and $S_{c}$ are measures of the excess or deficit of solute supply (diffusive or convective) relative to solute kinetics in the M-CELS (diffusive or consumptive).

### 2.6. Numerical methods

The simulations of coupled fluid flow and nutrient transport were implemented in the commercial software COMSOL Multiphysics^TM^ (COMSOL AB, Sweden, 6.1). The electronic supplementary material, Section M2 presents the details of that implementation and the dimensional parameter values.

### 2.7. Measures of solute transport

The goal of the simulations is to quantify the sufficiency of solute transport to cells in the M-CELS. That is measured by considering the shape and the surface area of the subset of $\Omega_{2}$ where $c_{2}$ is sufficiently high. The boundary of sufficient transport (or necrotic boundary if the solute is a nutrient) is defined as


(2.25) $$\Gamma_{n}=\{x_{2}|c_{2}=c_{n}\},$$

where $c_{n}$ is a threshold concentration that depends on the metabolic needs of the cells. We use $c_{n}=10^{-3}\;c_{0}$ for generality. The centre of mass of the zone of insufficient transport (or necrotic centre if the solute is a nutrient) has the coordinates


(2.26) $$\{{\bar{x}}_{n},{\bar{y}}_{n}\}=\left\{\frac{\int_{c_{2}<c_{n}}x_{2}\;dA}{\int_{\Omega_{2}}dA},\frac{\int_{c_{2}<c_{n}}y_{2}\;dA}{\int_{\Omega_{2}}dA}\right\}.$$

The fraction of sufficient transport is defined as the fraction of cells receiving solutes at a concentration above $c_{n}$. It is termed the live fraction if the solute is a nutrient and is defined as


(2.27) $$\Phi_{L}=\frac{\int_{c_{2}>c_{n}}dA}{\int_{\Omega_{2}}dA}.$$

For simplicity and without loss of generality, the terms associated with nutrients are used in the following sections.

### 2.8. Definition of asymptotes

**Figure 4.**
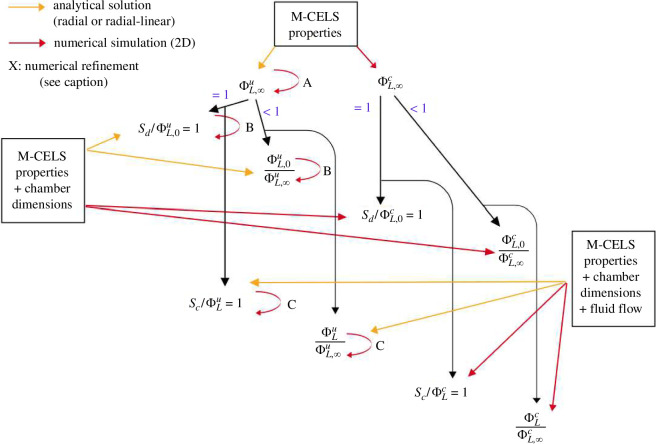
Flow chart of solute-transport analysis in M-CELS set in a culture chamber. Superscripts u and c respectively refer to ‘unconfined’ and ‘confined’. Capital letters refer to effects captured by the simulations but not by the analytical expressions. A: mathematical approximation and Michaelis–Menten consumption kinetics. B: two-dimensional effects, mostly the constriction between $\Omega_{2}$ and $W_{h}$. C: asymmetry of fluid flow in $\Omega_{1}$ and fluid-recirculation zones near the confinement corners.

The analysis is constructed as follows and depicted in figure 4. We first consider the effect of M-CELS confinement independently of solute supply, i.e. at $\gamma^{*}=1$. This is the maximal-supply asymptote and is indicated by an ∞ subscript. It is the theoretical maximum of solute transport that is achievable in a cell-culture device. We then consider the effect of finite diffusive transport in static culture, termed the static asymptote and indicated by a 0 subscript. We finally consider the role of convective supply, i.e. the trajectory that $\Gamma_{n}$ and $\Phi_{L}$ follow between their static and maximal-supply asymptotes as convective supply increases. The maximal-supply asymptote determines whether sufficient transport is achievable in the whole M-CELS. If that is the case, any insufficiency is termed supply-induced, and a condition is derived for its prevention. If not, insufficiency is termed rate-induced.

## 3. Results

### 3.1. Maximal-supply asymptote

At the radially symmetric maximal-supply asymptote, the $C^{1}$-regularity of $c_{2}$ implies that $c_{2}$ is zero with a zero gradient at the necrotic radius $r_{n}$. This introduction of $r_{n}$ has been done by others [19] and was shown to satisfactorily approximate Michaelis–Menten kinetics if $K_{1/2}^{*}\ll 1$ [29]. This is generally verified for cellular oxygen consumption. The solution of the transport equation (2.9) approximated with a constant consumption rate yields the following equation for $r_{n}^{*}=r_{n}/a$ (details in electronic supplementary material, Section R1):


(3.1) $$1=\frac{Da}{4}\left(1-r_{n}^{*2}\right)+\frac{Da}{2}r_{n}^{*2}\ln(r_{n}^{*}).$$

**Figure 5.**
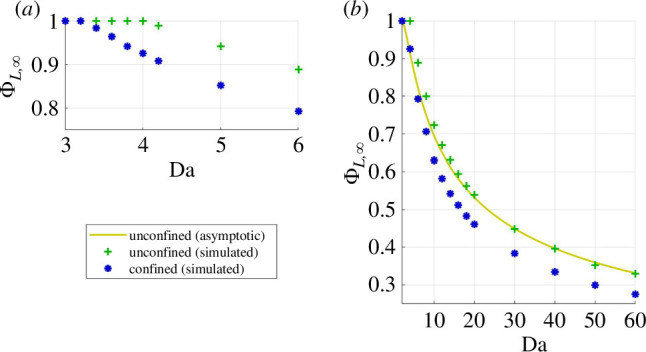
Live fraction at maximal supply (*a*) around the onset of necrosis, which occurs at lower Da in confinement, and (*b*) closely matching its asymptotic expression for Da>10 (error under 5%).

If $r_{n}^{*}\ll 1$, then $-r_{n}^{*2}\ln(r_{n}^{*})\gg r_{n}^{*2}$. Equation (3.1) simplifies into a second-order polynomial of $r_{n}^{*}\sqrt{-\ln(r_{n}^{*})}$. It admits a real solution if Da≥4, which is positive and tends to 0 when Da⟶4. Let ${Da}_{\infty }=4$ be the onset of rate-induced necrosis. The numerical simulations verified this in the unconfined geometry ($\Phi_{L,\infty }<1$ for Da>4) and showed ${Da}_{\infty }=3.4$ in the confined geometry (figure 5*a*). The penalization of cell survival by confinement translates into the maximum non-necrotic radius being 92% of the equivalent unconfined M-CELS.

If the necrotic area is large, $r_{n}^{*}\approx 1$ and Da≫1. Let $\delta^{*}=1-r_{n}^{*}$. The logarithm term may be expanded around $\delta^{*}=0$ as


$$\begin{array}{rl} \ln\left(r_{n}^{*}\right) & =\ln\left(1-\delta^{*}\right)\\ & =-\delta^{*}-\frac{\delta^{*2}}{2}, \end{array}$$

which yields the following expansion of equation (3.1) at order 2 in $\delta^{*}$:


(3.2) $$\frac{Da}{2}\delta^{*2}-1=0.$$

The asymptotic expressions for $r_{n}^{*}$ and $\Phi_{L}^{\infty }$ at high Da follow:


(3.3) $$\begin{array}{rl} r_{n}^{*} & =1-{\left(\frac{2}{Da}\right)}^{1/2}, \end{array}$$

(3.4) $$\begin{array}{rl} \Phi_{L}^{\infty } & \;=2\left(-\frac{1}{Da}+{\left(\frac{2}{Da}\right)}^{1/2}\right). \end{array}$$

The latter is an excellent predictor of the unconfined live fraction for Da≥10 (error under 5%, figure 5*b*). The confined live fraction in the asymptotic high-Da regime was lower by around 15% for Da≥10. This translates into necrotic radii being around 9% larger than in the equivalent unconfined M-CELS.

**Figure 6.**
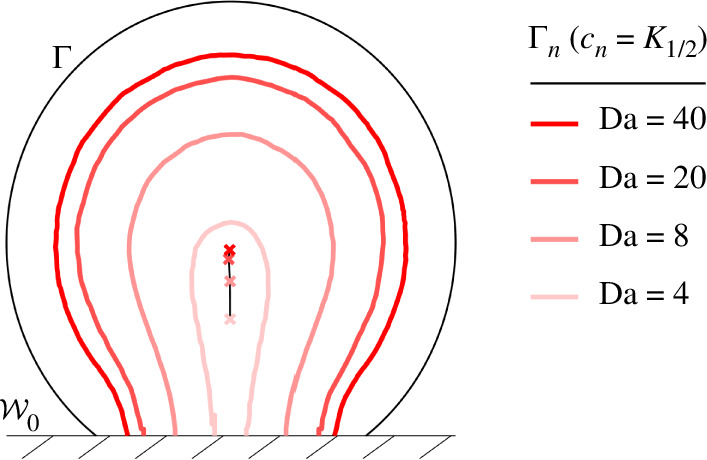
Necrotic boundaries $\Gamma_{n}$ at maximal supply in confined M-CELS, with necrotic tissue in the area enclosed by $\Gamma_{n}\cup W_{0}$. Necrosis grows perpendicularly from the confining surface for Da∼1 and increases before expanding in all directions at higher Da. Positions of necrotic-core barycentres marked by ×.

Figure 6 shows the shape of necrotic cores in confined M-CELS for varying Da. The contact surface skews the cores towards it. Those appear along the surface’s normal bisector when Da becomes larger than ${Da}_{\infty }$. Necrosis encompasses the M-CELS barycentre from its onset (see $\Gamma_{n}$ at Da=4). The normal to the contact surface passing by the barycentre thus is the region most vulnerable to necrosis.

### 3.2. Static asymptote

Let us obtain an analytical approximation of $\Phi_{L}^{0}$ on the quasi-one-dimensional domain $\hat{\Omega }$. The non-dimensional necrotic radius ${\hat{r}}_{n}^{*}={\hat{r}}_{n}/a$ satisfies this equation,


(3.5) $$-\frac{1}{S_{d}}(1-{\hat{r}}_{n}^{*2})+1=\frac{Da}{4}(1-{\hat{r}}_{n}^{*2})+\frac{Da}{2}{\hat{r}}_{n}^{*2}\ln({\hat{r}}_{n}^{*}),$$

whose derivation is detailed in electronic supplementary material, Section R2.

#### 3.2.1. Prevention of necrosis

Let us investigate the prevention of supply-induced necrosis at $Da<{Da}_{\infty }$. When the necrotic core is small, $|\;\ln({\hat{r}}_{n}^{*})|\gg 1$, so (3.5) is approximated as


(3.6) $$-\frac{Da}{2}{\hat{r}}_{n}^{*2}\ln({\hat{r}}_{n}^{*})=\frac{1}{S_{d}}+\frac{Da}{4}-1.$$

This admits a real solution if the right-hand term is positive. The necrotic core vanishes when that term becomes negative, i.e. with the unconfined ${Da}_{\infty }=4$,


(3.7) $$S_{d}\left(1-\frac{Da}{{Da}_{\infty }}\right)\geq 1.$$

This satisfies physical intuition about extreme values of Da. When Da is low, it suffices that as many nutrients arrive at the interface as the M-CELS consumes ($S_{d}>1$). As Da increases, $\gamma^{*}$ needs to become ever closer to 1 to prevent supply-induced necrosis, which requires an excess of nutrient supply and $S_{d}>1$. Finally, the value of $S_{d}$ required for necrosis prevention diverges as Da approaches the onset of rate-induced necrosis. The condition (3.7) is expressed using the velocity scale of diffusion, which is $v_{\mathrm{dif}}=2D/d$ at a diffusivity D across a domain of length d,


(3.8) $$v_{dif,1}\geq \frac{v_{dif,2}}{2\left(\frac{1}{Da}-\frac{1}{{Da}_{\infty }}\right)}.$$

Necrosis prevention by high diffusive solute supply is limited by fast diffusion in the M-CELS. Preventing necrosis indeed requires raising the concentration on the fluid–M-CELS interface and that concentration is lower when the diffusive-flux balance favours transport away from the interface, into the M-CELS. The diffusive-supply requirement is more stringent for small M-CELS with a fast metabolism than for large M-CELS with a slow metabolism. This consideration applies to oxygen, whose diffusivity is of the order of $10^{-9}\;m^{2}\;s^{-1}$ in tissues but whose consumption rate varies over two orders of magnitude between cell types ([20,31]).

The design constraints of experimental set-ups in practice lower the onset of rate-induced necrosis. They include space constraints on microscope stages and, often, room for the culture of multiple M-CELS. These both impose a minimal value on L of at least a few millimetres. Consequently, considering that $D_{2}/D_{1}$ is typically between 0.01 and 1, $R_{d}$ is constrained under 10, and under 1 for nutrients like oxygen which readily cross cell membranes and for which $D_{2}/D_{1}\approx 1$. For oxygen, the maximal Da at which adequate nutrient supply can be achieved in static culture is Da≈1. There, the condition (3.7) implies $R_{d}>2/3$, which is close to its limit.

**Figure 7.**
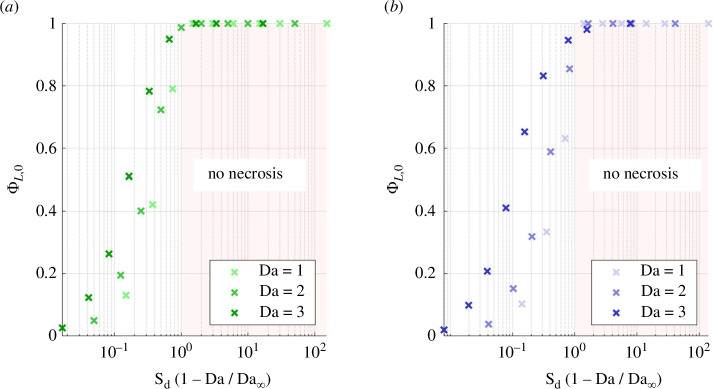
Live fraction at the static asymptote, for Da≤3 and $R_{d}$ varying between 0.1 and 100, in the (*a*) unconfined and (*b*) confined geometry. It suffices that the excess of diffusive solute supply satisfies $S_{d}(1-Da/{Da}_{\infty })>M$ for supply-induced necrosis to be prevented, with M=1 in the unconfined geometry and M=2 in the confined one.

The condition (3.7) is verified by numerical simulations of the unconfined geometry (figure 7*a*). Necrosis prevention is similarly achieved in the confined geometry, with $S_{d}(1-Da/{Da}_{\infty })>1$ and ${Da}_{\infty }=3.4$ (figure 7*b*). An approximately twice higher diffusive flux to Γ is required to prevent necrosis relative to the unconfined geometry. This corresponds to a reduction of the maximal permissible distance between a solute reservoir and an M-CELS by half in static culture, if all other parameters are equal.

#### 3.2.2. Approach of maximal-supply asymptote

Let us investigate the live fraction as a function of diffusive supply when there is rate-induced insufficient transport and diffusive supply is ample, i.e. $S_{d}\gg 1$, and $R_{d}\gg 1$. The live fraction normalized by its maximal-supply value is analytically derived in electronic supplementary material, Section R3 as


(3.9) $$\frac{\Phi_{L}^{0}}{\Phi_{L}^{\infty }}=-(R_{d}S_{d}{)}^{-1/2}+{\left(1+(R_{d}S_{d}{)}^{-1}\right)}^{1/2}.$$

This describes a sigmoid function of $S_{d}$ with an inflection point—the point where $\Phi_{L}^{0}=\Phi_{L,\infty }/2$—shifted by $R_{d}$. As for necrosis prevention, the excess of diffusive supply relative to consumption needs to be larger when diffusion into the M-CELS is supply-limited. The variable $R_{d}S_{d}$ may be written


(3.10) $$R_{d}S_{d}=\frac{2c_{0}D_{1}^{2}}{R_{max}L^{2}D_{2}}.$$

Counter-intuitively, in static culture, the proximity of an M-CELS with high Da to its maximal-supply asymptote does not depend on the M-CELS radius. It therefore does not depend on the M-CELS age if transport properties remain constant. The expression (3.9) also informs on how to adapt experimental parameters ($c_{0}$ and L) based on those that may evolve with M-CELS development and health ($R_{\max}$ and $D_{2}$). For example, the tight alignment of neurons of the outer cell layer of cortical-brain organoids [32] increases $D_{2}$ because the tortuosity becomes close to 1. If M-CELS diffusivity becomes $D_{2}^{\prime }=\lambda D_{2}$, solute concentration needs to become $c_{0}^{\prime }=\lambda c_{0}$ or the distance between the solute source and $\Omega_{2}$ needs to become $L^{\prime }=\lambda^{-2}L$ to maintain solute penetration.

**Figure 8.**
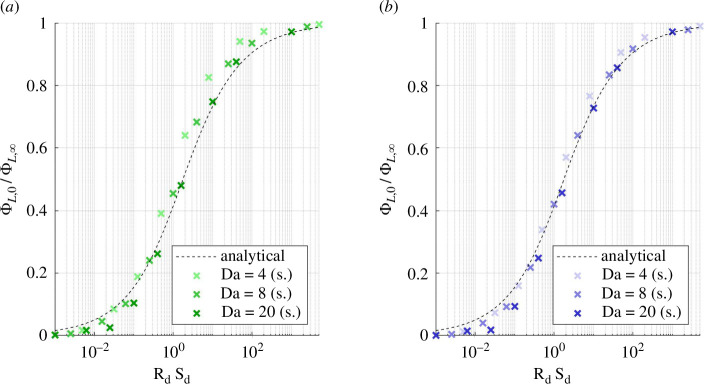
Live fraction at the static asymptote for Da≥4, where rate-induced necrosis occurs. (*a*) Unconfined and (*b*) confined geometry. Good agreement with the analytical expression (3.9) from the quasi-one-dimensional domain $\hat{\Omega }$, especially at large diffusive supply ($R_{d}S_{d}>1$). s.: simulated.

Equation (3.9) is a good predictor of the simulated live fraction for Da≥4 in both the unconfined and confined geometries (figure 8). It overestimates the live fraction at low $S_{d}$ because it does not account for the constriction between Γ and $W_{h}$. Solutes at low $S_{d}$ only reach the parts of $\Omega_{2}$ closest to I and O, so the uniform-γ assumption is less accurate. The live fraction progressed towards its maximal-supply asymptote along a similar trajectory in the confined geometry but with a delay. $S_{d}$ needed to be approximately 50% higher for the relative live fraction to progress to the same stage as if unconfined.

At the static asymptote, the distance between the solute reservoir and the M-CELS implies that supply to the M-CELS surface is reduced. Preventing necrosis for Da close to the onset of rate-induced necrosis requires an excess of diffusive nutrient supply relative to nutrient consumption that is experimentally hard to achieve. The practical maximum Da in static culture can be lower than 1, which means a division by 2 of the maximum non-necrotic radius compared with maximally supplied M-CELS. For M-CELS with rate-induced necrosis, the distance to the maximal-supply asymptote does not depend on their radius, so the efficacy of solute supply in a no-flow set-up is constant for M-CELS with age-independent transport properties.

### 3.3. Microfluidic culture

#### 3.3.1. Function of increased convective supply

**Figure 9.**
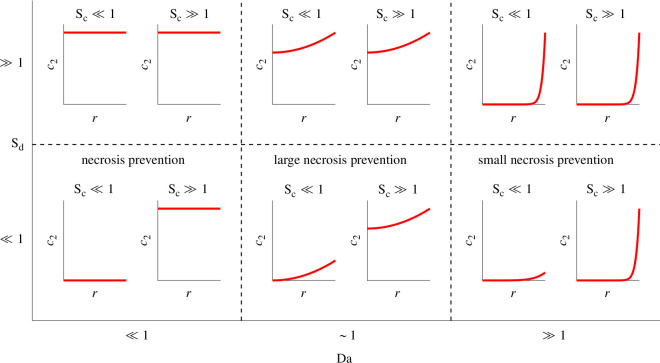
Qualitative effect of increased convective nutrient supply to $\Omega_{2}$. For deficient diffusive supply (low $S_{d}$), an ample convective supply may prevent supply-induced necrosis at low Da ($Da<{Da}_{\infty }$), but cannot prevent rate-induced necrosis and would only marginally increase nutrient supply to the outer cell layers at $Da\gg {Da}_{\infty }$.

Convective solute supply only affects transport in the M-CELS if there is a deficiency at the static asymptote, i.e. $S_{d}≲1$. Then, convection in $\Omega_{1}$ fulfils three distinct functions depending on Da (figure 9). If Da≪1, concentration in $\Omega_{2}$ is approximately uniform. Any necrosis is supply-induced and can be prevented. It suffices that $S_{c}>1$. If Da≫1, most of the M-CELS is affected by rate-induced necrosis. External convection would increase solute supply only to cells that are closest to Γ. The penetration distance of solutes would increase at most until its value at the unconfined maximal-supply asymptote $\sqrt{2/Da}$, which still is poor transport. If Da∼1, necrosis may be supply-induced or rate-induced, according to a threshold that depends on M-CELS confinement. Analogously to the static asymptote, supply-induced necrosis can be prevented by $S_{c}(1-Da/4)≳1$. Rate-induced necrosis cannot be prevented but may be reduced by high $S_{c}$. External convection reduces the distance across which solutes need to diffuse to reach Γ, so it needs to increase when diffusive transport across areas of low fluid velocity is slow. Those areas are the diffusive boundary layer over Γ and the vicinity of the confinement corner ($\Gamma \cap \Omega_{2}$). Since $R_{d}S_{d}$ characterizes the efficacy of diffusive supply, we postulate that $S_{c}∼(R_{d}S_{d}{)}^{-1}$ to have a microfluidic system approach the maximal-supply asymptote.

#### 3.3.2. Evolution of solute supply with increasing convection

**Figure 10.**
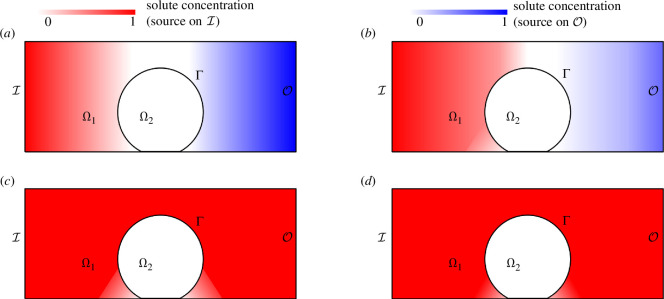
Schematic evolution of concentration in $\Omega_{1}$ under increasing convection from I to O. (*a*) Diffusive equilibrium with a symmetric concentration around {x=0}. (*b*) Low convection ($S_{c}∼1$) increases concentration on the upstream side of $\Omega_{2}$ but decreases it on its downstream side. The upstream increase is limited near the confinement corner. (*c*) Convection of higher magnitude ($S_{c}\gg 1$) fills the primary flow region in $\Omega_{1}$ with solute (c≈1 there), but not the confinement corners. (*d*) At ever higher convection, concentration increases in the confinement corner due to increased corner-eddy intensity.

Let us describe how progressively higher convection in $\Omega_{1}$ affects solute supply to Γ and its concentration in $\Omega_{2}$. At the static asymptote, solutes diffusing from I and O form a symmetric concentration field around $\Omega_{2}$ (figure 10*a*). Low convection from I to O (${Pe}_{1}>1$ but $S_{c}<1$) enhances solute transport from I but inhibits it from O (figure 10*b*). The interface concentration γ increases on the upstream side of Γ but decreases on its downstream side. The area of insufficient supply is shifted towards O. The supply increase on the upstream side of Γ mostly concerns its portion that is not affected by the confinement corner. Fluid flow near the latter indeed occurs as an infinite sequence of recirculation eddies of decreasing sizes and intensities [33]. Those were observed in the confined geometry because of the very large resistance to flow of $\Omega_{2}$, even though fluid transpires across Γ. Let $\Gamma_{\rho }$ be the subset of Γ exposed to the recirculation eddies (electronic supplementary material, figure S4). Solute transport in that region is diffusion-dominated unless the convection in $\Omega_{1}$ is very large (the maximum velocity on the recirculation eddies decreases geometrically, with a ratio of approximately$\;10^{-3}$ for the 60° angle of the confined geometry [33]). The efficacy of static solute supply thus remains determinant for the regions of $\Omega_{2}$ near the confinement corner even when transport is convection-dominated in most of $\Omega_{1}$. As convection further increases ($S_{c}>1$), the solute flux from I becomes sufficient to fill the supply deficiency on the downstream side of Γ exposed to the primary flow and any remaining necrosis again becomes symmetric about x=0 (figure 10*c*). Solute supply increases to the confinement corner as convection further increases and transport in the largest recirculation eddy eventually becomes convection-dominated (figure 10*d*).

#### 3.3.3. Prevention of necrosis

In the quasi-one-dimensional domain $\hat{\Omega }$, the non-dimensional necrotic radius ${\hat{r}}_{n}^{*}={\hat{r}}_{n}/a$ satisfies the following equation:


(3.11) $$\frac{e^{-{Pe}_{1}}-1}{S_{c}}(1-{\hat{r}}_{n}^{*2})+1=\frac{Da}{4}(1-{\hat{r}}_{n}^{*2})+\frac{Da}{2}{\hat{r}}_{n}^{*2}\ln({\hat{r}}_{n}^{*}),$$

whose derivation is detailed in the electronic supplementary material, Section R5. Let us assume that convection is sufficiently high that $c_{2}$ is approximately symmetric and that equation (3.11) may be used on Ω.

The necrosis-prevention condition on $S_{c}$ is obtained from the same reasoning as for equation (3.7) at the static asymptote. Noting that $-1<e^{-{Pe}_{1}}-1<0$, it suffices that


(3.12) $$S_{c}(1-Da/4)>1.$$

In terms of peak inlet fluid velocity, which is the most easily adjustable experimental parameter, this condition can be written


(3.13) $$v_{0}>\frac{v_{dif,2}}{4\left(\frac{1}{Da}-\frac{1}{4}\right)}.$$

This depends on M-CELS properties only and not on diffusive transport in the microfluidic chamber. Like at the static asymptote, this condition expresses that necrosis prevention is penalized by fast diffusion in the M-CELS.

For an unconfined M-CELS of radius 0.5 mm where Da=3 characterizes oxygen kinetics, the inlet-velocity condition is $v_{0}∼10^{-5}$ m s^−1^. The corresponding inlet flow rate for an M-CELS culture chamber of 1 cm × 1 mm cross-section (e.g. [17,18]) is 1 ml min^−1^. Flow rates must be limited by the physiological shear stress of the cultured cells, which is under 10^−3^ Pa at rest outside of blood vessels [34]. In order not to affect cell function in an M-CELS, peak microfluidic velocity thus should remain under 10^−4^ m s^−1^ (for a fluid viscosity of 1 mPa s and a gap of 100 between M-CELS apex and channel ceiling). That corresponds to 10 ml min^−1^ with a 1 cm × 1 mm cross-section. Necrosis prevention via external nutrient convection over an unconfined M-CELS is therefore experimentally achievable.

**Figure 11.**
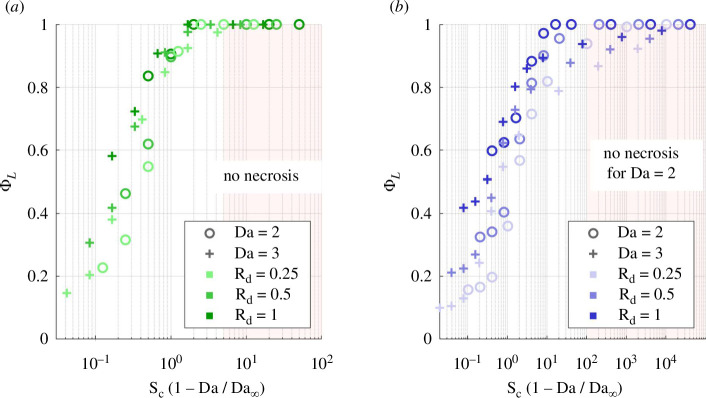
Simulated live fraction in microfluidic culture for Da such that any necrosis is supply-induced ($Da<{Da}_{\infty }$). (*a*) *Unconfined geometry*: necrosis prevention achieved if $S_{c}(1-Da/4)>5$ for Da=2 and Da=3. (*b*) *Confined geometry*: no excess of convective supply suffices for general necrosis prevention. The required excess increases with a more deficient diffusive supply, i.e. with lower $R_{d}$ and higher Da.

Numerical simulations show that necrosis prevention is achieved if $S_{c}(1-Da/4)>5$ in the unconfined geometry (figure 11*a*). The difference with the analytical condition (3.12) results from the diffusive boundary layer that grows from Γ into $\Omega_{1}$, which nutrients need to cross to reach $\Omega_{2}$. In the confined geometry, nutrients also need to diffuse across the corner recirculation areas, so a much higher excess of convection is required to prevent necrosis. That excess increases with lower $R_{d}$ and higher Da, such that $S_{c}∼10^{4}$ is insufficient to prevent necrosis at Da=3 (figure 11*b*). This value is not advisable in experiments because it implies unphysiological shear stress on cells. For oxygen in a confined M-CELS of radius 0.5 mm, the convection limit due to shear stress is at $S_{c}∼100$. This means that necrosis prevention by microfluidic flow is possible if $R_{d}\geq 0.5$ at Da=2, but impossible if insufficient diffusive supply means that it exists at Da=3.

#### 3.3.4. Transport asymmetry under moderately high convection

**Figure 12.**
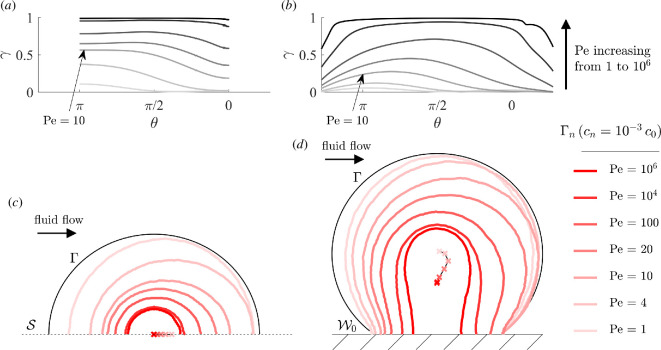
Solute concentration in $\Omega_{2}$ in microfluidic culture, example of Da=5, $R_{d}=0.5$ (above the onset of rate-induced necrosis, with supply-limited diffusion into $\Omega_{2}$). Varying convection, ${Pe}_{1}$ between 1 and $10^{6}$. Moderately high convection (here, ${Pe}_{1}∼1$ to ${Pe}_{1}∼100$) induces asymmetry in solute supply and in necrotic cores in the direction of convection. Confinement induces a delay in the increase in solute supply near the confinement corners. (*a*) Concentration on Γ, unconfined geometry and (*b*) confined. (*c*) Necrotic boundaries $\Gamma_{n}$, unconfined geometry and (*d*) confined.

The asymmetry of solute supply to Γ when ${Pe}_{1}>1$ and $S_{c}<1$ translates into asymmetric necrotic cores, as the example of Da=5 and $R_{d}=0.5$ shows (figure 12). The shape of the cores follows the progressive supply increase to Γ as convection in $\Omega_{1}$ increases. The cores first shrink on the upstream half of $\Omega_{2}$ (Γ∩{x<0}), before shrinking along y as the supply deficiency is filled in the constriction between Γ and $W_{h}$, and finally shrinking on the downstream half of $\Omega_{2}$. In the confined geometry, the third phase is followed by a reduction of the cores near the confinement corners. In that phase, ever-increasing convection in the primary flow region increases the intensity of the first recirculation eddy and therefore convective solute fluxes near the parts of Γ exposed to the recirculation region (figure 12*b,d*).

To estimate the configuration of maximum asymmetry of solute concentration, we consider the quasi-one-dimensional domain $\hat{\Omega }$ and derive the conditions where ${\hat{\gamma }}^{*}=0.5$ (electronic supplementary material, Section R6). Those are


(3.14) $${Da}^{1/2}S_{c}=4\sqrt{2}.$$

**Figure 13.**
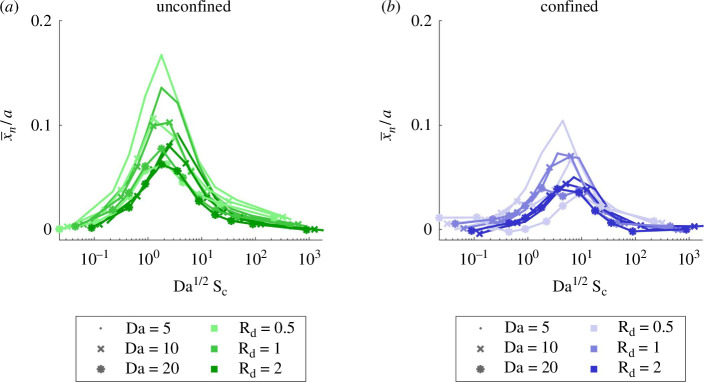
Trajectory of necrotic barycentres in microfluidic culture (x-coordinate). The value of ${Da}^{1/2}S_{c}$ determines an asymmetry peak. (*a*) Unconfined geometry and (*b*) confined.


The quantity ${Da}^{1/2}S_{c}$ is a good predictor of the maximum asymmetry of simulated necrotic cores (figure 13). Using dimensional parameters,

(3.15) $${Da}^{1/2}S_{c}=2v_{0}\sqrt{\frac{c_{0}}{R_{max}D_{2}}}.$$

The symmetric nature of solute concentration thus only depends on solute kinetics inside M-CELS and on boundary conditions on I. Since it does not depend on the M-CELS diameter or the microfluidic channel dimensions, the peak inlet velocity could be used as a single parameter to induce persistent asymmetric concentrations inside M-CELS and investigate directional responses. The maximum downstream shift of the simulated necrotic barycentres occurs for ${Da}^{1/2}S_{c}\in \left[1,4\right]$ in the unconfined geometry (figure 13*a*), showing that ${Da}^{1/2}S_{c}$ is a good predictor of supply asymmetry. The shift was delayed and less marked in the confined geometry because solute supply increases more slowly near the recirculation areas: its maximum occurs for ${Da}^{1/2}S_{c}\in \left[3,10\right]$ (figure 13*b*).

#### 3.3.5. Approach of maximal-supply asymptote

Let us consider cases of rate-induced necrosis ($Da>{Da}_{\infty }$) and assume that convection is sufficiently high that the asymmetry peak has passed. We consider this to be ${Da}^{1/2}S_{c}>8$ in the unconfined geometry and ${Da}^{1/2}S_{c}>20$ in the confined geometry, i.e. twice the asymmetry peak. The approach of the maximal-supply asymptote depends on the rate at which solute supply increases on Γ via the diffusion-dominated areas. Let us consider the recirculation areas and define a corner Péclet number ${Pe}_{\rho }$ based on the recirculation properties and the quasi-one-dimensional no-flow solution,


(3.16) $${Pe}_{\rho }=\frac{\gamma v_{\rho ,1}}{D_{1}{\frac{dc}{d\hat{x}}|}_{\hat{\Gamma }}},$$

where $v_{\rho ,1}$ is the maximum velocity of the largest eddy, with $v_{\rho ,1}=qv_{0}$ and $q∼10^{-3}$ (fig. 5 in [33]). From the no-flow expression of concentration in $\hat{\Omega }$, obtained by substituting electronic supplementary material equations (22) into (20), we obtain


(3.17) $${Pe}_{\rho }=q{Pe}_{1}\left(\frac{S_{d}}{1-{\hat{r}}_{n,0}^{*2}}-1\right).$$

Substituting ${\hat{r}}_{n,0}^{*}$ with $\delta_{0}^{*}$ using electronic supplementary material, equation (29) and expanding at order 1 in $\delta_{0}^{*}$ gives


(3.18) $${Pe}_{\rho }=q{Pe}_{1}\left(\frac{R_{d}S_{d}}{2(\sqrt{1+R_{d}S_{d}})-1}-1\right).$$

Under deficient diffusive supply ($R_{d}S_{d}\ll 1$), successive Taylor expansions lead to


(3.19) $${Pe}_{\rho }=qR_{d}S_{c}.$$

The balance between convective supply and consumption on $\Gamma_{\rho }$ is defined, analogously to $S_{c}$, as


(3.20) $$\begin{array}{rl} S_{c,\rho } & =\frac{2R_{d}{Pe}_{\rho }}{Da}\\ & =qR_{d}S_{d}S_{c}. \end{array}$$

A similar reasoning may be followed for the diffusive boundary layer covering all of Γ. The velocity of the first eddy $qv_{0}$ would be replaced by the velocity at the entrance of the boundary layer, which also scales with $v_{0}$. Solute supply to Γ thus is governed by $R_{d}S_{d}S_{c}$ when $S_{c}\gg 1$. The excess of convective supply required to maintain a given proportion of live cells ($\Phi_{L}$) scales with $(R_{d}S_{d}{)}^{-1}$. The corresponding peak inlet fluid velocity scales like


(3.21) $$v_{0}∼\frac{v_{dif,2}}{S_{d}^{2}}.$$

The approach of maximal supply via external convection thus is delayed by deficient diffusive supply to the M-CELS and by fast diffusion within it. Using dimensional parameters,


(3.22) $$v_{0}∼aD_{2}{\left(\frac{R_{max}}{c_{0}}\right)}^{2}{\left(\frac{L}{D_{1}}\right)}^{2}.$$

This velocity evolves linearly with the M-CELS diameter, so it would need to increase with M-CELS age if solute penetration distance were to be maintained for an experiment. In the example of neurons in a cortical organoid, the increase in radial diffusivity resulting from neuronal alignment would also contribute to $v_{0}$ needing to increase with M-CELS age. The dependency of $v_{0}$ on the square of the inverse of the inlet concentration and the square of the distance to the solute source provides a means to reduce experimentally required flow rates. These come with the caveats that inlet concentrations are often physically or biologically constrained (e.g. dissolved gases like oxygen have a saturation limit) and that the distance to the solute source is constrained.

**Figure 14.**
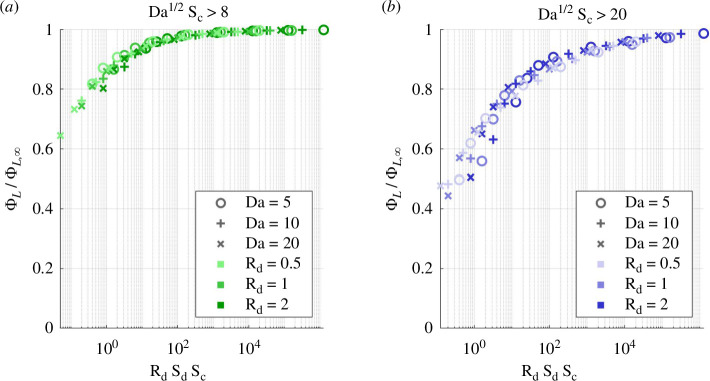
Simulated live fraction in microfluidic culture for $Da\geq {Da}_{\infty }$, where there is rate-induced necrosis. Live fraction normalized by its maximal-supply value. The approach of the maximal-supply asymptote is governed by $R_{d}S_{d}S_{c}$. (*a*) Unconfined geometry and (*b*) confined.

The numerical simulations verify that the rate of approach of maximal supply is governed by $R_{d}S_{d}S_{c}$. In the unconfined geometry, overcoming the diffusive boundary layer on Γ to achieve $\Phi_{L}/\Phi_{L,\infty }=0.9$ requires $R_{d}S_{d}S_{c}>10$ (figure 14*a*). In the confined geometry, the additional diffusive barrier constituted by the corner recirculation areas make that condition $R_{d}S_{d}S_{c}>10^{3}$. This limits the biological configurations where supply deficiency can be filled within the constraints of shear stress on cells.

### 3.4. Comparison with previous models and experiments

The results presented above shed new light on some previously published simulations and experiments (table 2). Let us note that the predicted onset of necrosis in the unconfined geometry (${Da}_{\infty }=4$) is in agreement with calculations in cylinders in [20] and [29] and that spheres are characterized by ${Da}_{\infty }=6$, as these studies demonstrate. Similarly, the solute quantity balances on Γ are defined as $S_{d}=3R_{d}/Da$ and $S_{c}=3R_{d}{Pe}_{1}/Da$ for spheres.

**Table 2.** Application of results to previous studies. Subscripts 1 and 2 are applied to the fluid or gel outside the M-CELS of interest and to the M-CELS, respectively (as in the models). Details of the values used for the comparisons are in the electronic supplementary material, table S3.

M-CELS type	confining geometry	study results^a^
human midbrain organoid [15]	adhesion to bottom of reservoir and encapsulation in GelTrex	10% reduction in necrotic area under continuous fluid flow vs. a shaking platform (from 16% to 14%).
Application: Da=0.5 for oxygen in the 400 µm diameter spherical organoids. The 1 mm thick gel capsule imposes a diffusion-limited zone which can be represented by $S_{d}=0.75$ if fluid flow is large enough that $c_{1}^{*}=1$ outside of it. $\Phi_{L}$ is very sensitive to $S_{d}$ in that region, all the more so as Da is low, so it is coherent that necrosis would be large enough to stop growth for such a value of $S_{d}$ (figure 7*b*). Experimentally, $\Phi_{L}=0.85$. The model (not simulated for Da=0.5) would slightly underestimate that as there are quiescent cells near the necrotic area that consume less oxygen and thus induce deeper oxygen penetration than the model predicts.		
human retinal organoid [35]	ellipsoidal well, separated from fluid flow by a membrane	fluid flow increases the simulated penetration of oxygen and divides the experimental number of dead cells (Caspase3+) by 2.5.
Application: Da=4 for oxygen in the spherical 400 µm diameter organoids. In static conditions, $S_{d}=0.3$ and the model predicts^b^ $\Phi_{L,0}=0.4$. The distance between the organoid and the membrane implies that $S_{d}=1.5$ at best if fluid flows fast enough in the upper channel that $c_{1}^{*}=1$ there^c^. This would leave $\Phi_{L}=0.9$ under fluidic conditions and there would indeed be some Caspase3+ cells left. The given Caspase3+ ratio rather suggests $\Phi_{L}=0.75$, which corresponds to figure 11*b* with the experimental^d^ Da=4, $R_{d}=0.4$, and Pe=20.		
HepG2 tumour spheroid [36]	cylindrical well, separated from fluid flow by membrane	spheroids grow to a maximum diameter of 200 µm in static culture and to at least 300 µm in fluidic culture
Application: the maximum diameter corresponds to Da=1.6 and $S_{d}=0.13$ for oxygen, for which the model predicts $\Phi_{L,0}=0.2$ (figure 7*b*)^e^. Quiescent cells make this an underestimation, but it does indicate a large necrotic area which could be sufficient to stop growth [14]. In fluidic culture, $S_{c}∼10^{2}$ which is ample enough to avoid necrosis until spheroid diameters of at least 300 µm, as the authors observe.		

^a^ Only results that are traceable to the supply of a solute to M-CELS. Cell activity markers are therefore not considered here.

^b^ We assume that a sphere and a cylinder behave identically if they have the same Da/Da∞ and we follow the Da = 3 curve on figure 7.

^c^ *S*_d_ is calculated from the chip inlet in the static case and from the membrane in that hypothetical case where the upper channel is maximally supplied.

^d^ Da = 3 on the figure, see note b.

^e^ Da = 1 on the figure, see note b.

## 4. Discussion

Multi-cellular engineered living systems (M-CELS) are situated at a certain distance from the source of soluble nutrients, growth factors or drugs that need to be supplied to them. Since solute transport is diffusion-dominated within M-CELS, their internal solute distribution is linked to their external solute transport via the solute concentration at the fluid–M-CELS interface only. It thus is key to rapidly cross the source–M-CELS distance. Both slow external transport relative to M-CELS metabolic needs and a large surface area of contact with the culture-chamber walls limit solute penetration into M-CELS.

We quantified the maximum consumption-to-diffusion kinetics ratio (noted ${Da}_{\infty }$) that an M-CELS can reach without necrosis as a function of its structural confinement. Finite transport kinetics in microfluidic chambers imply the need for an excess of solute supply to the M-CELS surface. That excess is a hyperbolic function of the ratio $Da/{Da}_{\infty }$. The cost of preventing necrosis thus diverges when Da approaches ${Da}_{\infty }$, e.g. requiring infinite flow rates. This renders the realization of a non-necrotic M-CELS at ${Da}_{\infty }$ unfeasible and places a lower limit on the maximum consumption-to-diffusion ratio that can be achieved in a given experimental set-up. That limit depends on the design constraints of the set-up and cellular sensitivity to shear stress.

External convection brings about a higher solute supply to the fluid–M-CELS interface only after the solutes have crossed zones where their transport is diffusion dominated. These cover the whole fluid–M-CELS interface and include the diffusive boundary layer that grows from the interface into the fluidic chamber and the recirculation area near each confinement corner. That area exists at the intersection of the M-CELS and the wall in the cylindrical geometry we simulated. It would be present near any corner of angle inferior to 146° in the confinement structure of a spherical M-CELS [33]. Slow diffusion across those areas relative to solute consumption in the M-CELS limits the benefits of external convection. This arises when the diffusion-dominated areas are thick, e.g. when there are narrow corners, and when the diffusive supply in an equivalent static configuration is deficient, e.g. when the distance between the solute source and M-CELS is large. In both cases, slow diffusion kinetics in the culture chamber lower concentration at the fluid–M-CELS interface, and external convection needs to be all the higher to overcome the diffusive barrier. The same reasoning applies to M-CELS encapsulated in hydrogels. Solute diffusivity across hydrogels is close to their diffusivity in water because hydrogel porosities are high, but capsule thickness is detrimental to increased solute penetration via external convection. One should thus aim for capsules to be as thin as possible so as not to impede solute supply while maintaining the cell–matrix interactions and the protection against shear stress that these provide.

The easiest way to increase solute supply to fluid–M-CELS interfaces is to modulate transport velocities, either by design for diffusion (short distance between solute source and M-CELS) or by operation for convection (peak fluid velocity). The effect of both is penalized by fast diffusion within the M-CELS, independently of how the internal-diffusion kinetics compare with consumption and external-diffusion kinetics. Fast internal diffusion means that any increase in solute supply to the fluid–M-CELS interface is more rapidly distributed throughout the M-CELS, slowing down the increase in interface concentration that is necessary to increase solute penetration depth. Achieving non-necrotic M-CELS (or a given penetration depth) at a given Da thus requires less external convection in large M-CELS with low consumption than in small M-CELS with high consumption.

The fundamental understanding of how microfluidic chamber design and operation affect solute supply to M-CELS that we have presented allows us to state how much larger rigid avascular M-CELS can grow by adding fluid flow to the culture set-up. In the oxygen example that illustrated our results, the maximum no-flow Da was a quarter of ${Da}_{\infty }$, i.e. the maximum M-CELS radius was divided by 2. Fluid flow around an unconfined M-CELS allows to recover a maximal Da close to ${Da}_{\infty }$ (figure 11*a*). Peak fluid velocity is constrained in experimental set-ups because of the shear-stress sensitivity of cells, which capped the maximum achievable Da at ${Da}_{\infty }/2$ in the presence of recirculation areas, i.e. an increase in maximal radius of around 40% compared with static culture (figure 11*b*). Similarly, approaching maximal supply in an M-CELS with incomplete solute penetration required a 100 times faster fluid flow because of the need for solutes to cross the recirculation areas (figure 14*b*). This highlights the importance of eliminating thick diffusion-dominated areas in confinement structures. These exist in widely used U-shaped barriers and wells (e.g. [37]), but are absent from designs such as slit U-shaped wells [38] or porous confinement scaffolds [39]. These results also point to solute-transport benefits being inhibited by the encapsulation of M-CELS in hydrogels.

Real M-CELS culture systems contain three additional complexities that our models do not explicitly address. First, spheroids and many organoids (e.g. brain, liver) are spherical or close to it. That implies a larger ${Da}_{\infty }$ (6 for a sphere [20,29]), but the reduction in ${Da}_{\infty }$ by confinement and the shape of necrotic cores would be similar. In culture, spherical M-CELS receive solute from the third dimension in the same ways as from the one orthogonal to the inlet–outlet axis (y) in our two-dimensional models. Those are diffusion, whose fluxes are reduced by constriction, and convection from shear flow. The necrosis prevention conditions would therefore be the same within constants. Maximal-supply approach rates would be the same. Second, spherical M-CELS may be confined without recirculation areas, such as behind U-shaped traps with a slit [38]. This is an intermediary situation between the two studied here. The static and maximal-supply asymptotes would follow the same laws as in the confined geometry, but necrosis prevention and the approach of maximal supply by convection follow the unconfined geometry because they are not delayed by diffusion-dominated areas. Third, M-CELS may be cultured in arrays to increase experimental throughput, which is particularly beneficial on drug-testing platforms [8]. This format is detrimental to solute supply in static culture because of the increased distance to the solute source with each row of the M-CELS array. In fluidic culture, it suffices that the M-CELS are sufficiently far apart that solute mixing in the wake of the upstream M-CELS restores solute concentration in the fluid to its source value. We analyse concentration near an M-CELS in electronic supplementary material, Section R7 and show that the necrosis-prevention condition is transferable to an array of spheroids if the distance between spheroids is a tenth of the entry length of the culture channel, so typically of the order of 1 mm. This is more than the few hundred micrometres that often separate spheroids cultured in arrays [36,40,41]. Developing a predictive model of solute supply to arrays of M-CELS that incorporates the array dimensions would contribute to increasing the repeatability of assays performed on M-CELS. Biologically, the assumptions of uniform properties hold for tumour spheroids and early-stage spherical organoids. Later-stage ‘rough’ organoids, such as wrinkled brain organoids [32,42], do not because the troughs in their surface are diffusion highways that enable deeper penetration of solutes, particularly of fast-diffusing ones like oxygen and glucose. Organoids with differentiated cell layers, e.g. an outer layer of high-consumption neurons, would present a different maximal-supply asymptote [20], but the analysis of external supply would still apply to them. Current experimental efforts in organoid research focus on incorporating a perfusable vasculature to better mimic *in vivo* mechanical forces and solute-transport patterns. This study of the link between the geometry and operation of the culture set-up and solute concentration inside M-CELS can inform the boundary conditions at the entrance of the vessels. This would, in turn, contribute to quantifying the inhomogeneity of solute transport in perfusable M-CELS as well.

## Data Availability

The simulation results and processing codes are on the Zenodo repository [43]. Supplementary material is available online [44].
